# Rosacea scleritis and sclero-uveitis; under recognised and over treated

**DOI:** 10.1186/s12348-025-00546-x

**Published:** 2026-02-12

**Authors:** Josephine Richards

**Affiliations:** Department of Ophthalmology, Royal Perth Hospital, 197 Wellington St, Perth, WA 6000 Australia

**Keywords:** Ocular rosacea, Scleritis, Episcleritis, Sclero-uveitis, Uveitis, Cathelicidin, LL-17 hCAP-18, TLR2

## Abstract

**Background:**

Rosacea episcleritis, scleritis and uveitis may be missed when clinicians focus on the eye without appraising the adnexae and facial skin. Addressing rosacea can reduce treatment failures and avoid systemic complications of less effective or more toxic drugs.

**Case series and narrative review:**

This series presents four cases with scleritis and / or sclero-uveitis in the setting of facial or ocular rosacea. Where relevant, negative systemic scleritis work up is included. Common features were incomplete resolution or poor response to one or more of topical and systemic steroid, systemic non-steroidal anti-inflammatory, systemic immunomodulatory and topical antibiotic treatment. All cases responded promptly to treatment with oral doxycycline, allowing withdrawal of other treatment. A review of the literature is presented, updating understanding of immunogenetics and inflammatory pathways relevant to ophthalmic management of these cases. Important considerations include the role of steroids, vitamin D, microbes, toll like receptors and their influence on the cathelicidin (Ll-17) pathway in rosacea inflammation.

**Conclusion:**

Making a correct rosacea diagnosis and managing with doxycycline or related medication can result in rapid remission, minimising disease complications and side effects of less targeted drugs.

## Background

Rosacea is a heritable autoinflammatory disease with a prevalence of more than 10% in Northern European populations [1]. It may also be seen in people with darker skin where pigmentation makes the signs more difficult to recognise [2]. Ocular rosacea is seen mainly in adults. When it occurs in children, it is often missed or subject to delayed diagnosis as a result of being less common in this group [3, 4]. Literature review identifies rosacea scleritis and episcleritis only in large reviews and multicentre studies where it forms fewer than2% of reported cases [5, 6]. In an article describing epidemiology of episcleritis and scleritis in urban Australia in 2014-15, 230 cases were identified. A systemic cause was found in 10% of episcleritis and 34% of scleritis cases. No cases of rosacea related disease were recorded [7]. In the author’s Anglo-Celtic predominant Australian patient population, rosacea is an under-recognised cause of episcleritis and scleritis which is seldom identified prior to uveitis subspecialist referral. In this population, the author has also observed rosacea related post-operative uveitis to be associated with post-operative macular oedema which settles once the rosacea is adequately treated. Uveitis is outside the scope of the current discussion. Despite ocular, and sometimes facial features of rosacea, patients presented in this report have been referred to tertiary level ocular immunology services as a result of failure to respond to steroid, anti-inflammatory, immunosuppressive and antimicrobial treatment and some have experienced significant complications as a result of failure of therapy to target the underlying rosacea related immune drivers. All had a good response to and tolerance of doxycycline. With improved awareness, rosacea may feature more prominently as a cause of scleritis and episcleritis in future epidemiologic publications. Scleritis and episcleritis cases, once recognised as being rosacea related, may be rapidly and cost-effectively treated, potentially avoiding steroid and systemic immunomodulatory treatment.

## Case series

These cases were all referred into the tertiary ophthalmic care of ocular immunology trained consultants with expertise in management of systemic immunosuppression independent of rheumatology or immunology input. Case 1 with multiple immunologic treatment failures was managed with additional immunology interdisciplinary support. Following resolution of the acute inflammatory phase all patients received standard education about lid hygiene and warm compresses in conjunction with ongoing use of lipid-based lubricants. Compliance was suboptimal in patients 1, 2 and 4 who all relapsed. At the time these patients were seen, cyclosporine could be accessed only in compounded form in our health system at great expense so that it did not play the same role in management as it might in some situations where it is less expensive and more easily available. We are now able to use commercial brands but use is limited by high cost to the individual patient.

### Case 1

**Fig. 1:**
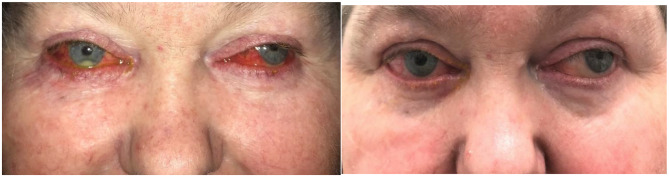
(Case 1): Left- Facial erythema and bilateral beefy red ocular injection and lid margin telangiectasiae, swelling and inflammation with right hypopyon following opening of Baerveld tube, 6 weeks after combined cataract-glaucoma surgery. Right- Reduced facial erythema and ocular injection with resolved hypopyon 10 days after starting doxycycline

A 61-year-old obese, hypertensive, diabetic woman with an exotropic blind left eye secondary to central retinal vein occlusion related neovascular glaucoma presented with bilateral mucopurulent conjunctivitis. She was on topical travoprost, brimonidine, timolol and brinzolamide for right ocular hypertension and left neovascular glaucoma. At the time of admission, she had diffusely injected aching eyes and culture grew methicillin resistant staphylococcus aureus. She also had moderate anterior chamber cells and flare with bilateral inferior pale granular keratic precipitates, prominent iris vascularity on the right and bilateral posterior synechiae. Standardised scleritis score (SSS) [8] was 10/25 Travoprost was switched to oral acetazolamide 125 mg twice daily. Topical moxifloxacin was commenced in conjunction with topical steroid drops. The mucopurulent discharge resolved within days but the scleral injection, pain and anterior chamber reaction persisted. A diagnosis of sclero-uveitis was made and the patient was treated with 3 days of pulsed intravenous methylprednisolone and thereafter prednisolone 60 mg. Negative or normal blood tests included ANCA, ANA, RF, CCP, syphilis serology and full blood count. Her inflammation improved on high dose oral and topical prednisolone but it was not possible to wean her down to a safe steroid dose: Methotrexate 15 mg was the maximum tolerable dose and was ineffective in allowing steroid weaning. Mycophenolate 3 g daily only allowed prednisolone weaning down to 20 mg daily. Her diabetic control worsened, she developed right cataract with vision loss from 6/9 at presentation down to counting fingers. Ocular hypertension progressed to glaucoma which could not be controlled medically. Nineteen months after presentation she was admitted for cyclophosphamide induction prior to cataract extraction and urgent implantation of a Baerveld tube. A culture negative purulent exudate developed along the suture line during the course of postoperative treatment with topical chloramphenicol and dexamethasone. When the tube opened 6 weeks post-operatively a hypopyon formed. There was no posterior segment inflammation and post-operative visual acuity of 6/12 was maintained. Anterior chamber tap was culture negative. For the first time her skin and adnexae were examined. Severe meibomitis, lid margin telangiectasiae, bilateral periocular erythema and mild facial rosacea were noted. Doxycycline 100 mg twice daily was commenced. Within 24 h the anterior chamber inflammation had improved and within a week both eyes and lids were white and quiet with SSS 1/25. Cyclophosphamide was ceased and prednisolone and mycophenolate were withdrawn over 4 months under doxycycline 50 mg daily cover. The sclero-uveitis remained in remission with uncorrected vision in the right eye 6/9 and moderate glaucomatous cupping on no topical treatment. Six months after ceasing cyclophosphamide treatment, renal cell carcinoma was diagnosed. Peri-operatively in the management of the renal tumour, doxycycline was stopped and the facial rosacea and scleritis recurred. Both settled within 10 days on 100 mg doxycycline twice daily and remission was maintained on ongoing oral therapy of 50 mg daily (Fig. 1).

### Case 2

A 71-year-old overweight man with cardiovascular disease and a history of recurrent marginal keratitis presented with severe deep red sectoral redness and scleral thickening (SSS 6/25) with subtle peripheral corneal infiltration without ulceration around an area of previous marginal keratitis. Chloramphenicol drops prescribed by his general practitioner in addition to his long-term lubricating drops had not helped. Ache was waking him at night. Naproxen 1 g slow release daily and topical dexamethasone helped partially with the pain but not the redness and corneal infiltrate. He was referred into the ocular immunology clinic for an opinion where his condition was labelled as sclerokeratitis. He had a mild rhinophyma, blotchy facial erythema and meibomian gland inflammation worse superotemporally around the involved eye. Doxycycline 100 mg twice daily was commenced and the sclero-keratitis, pain and facial redness improved dramatically within 10 days (SSS 0/25) following which naproxen was ceased. The doxycycline dose was dropped to 50 mg and continued till the end of 3 months. One subsequent mild recurrence of the scleritis responded promptly to doxycycline 100 mg daily reducing to 50 mg after 2 weeks.

### Case 3

**Fig. 2:**
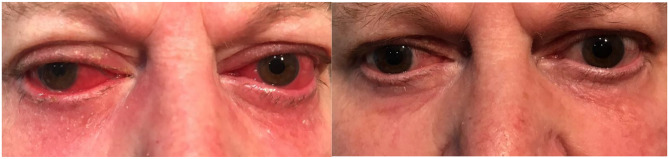
(Case 3): Left – bilateral mid-facial erythema, lid margin telangiectasiae, swelling and inflammation, ocular injection with right eye showing mucopurulent discharge. Right - Resolution of facial, lid and ocular surface inflammation 2 weeks after starting doxycycline

**Fig. 3:**
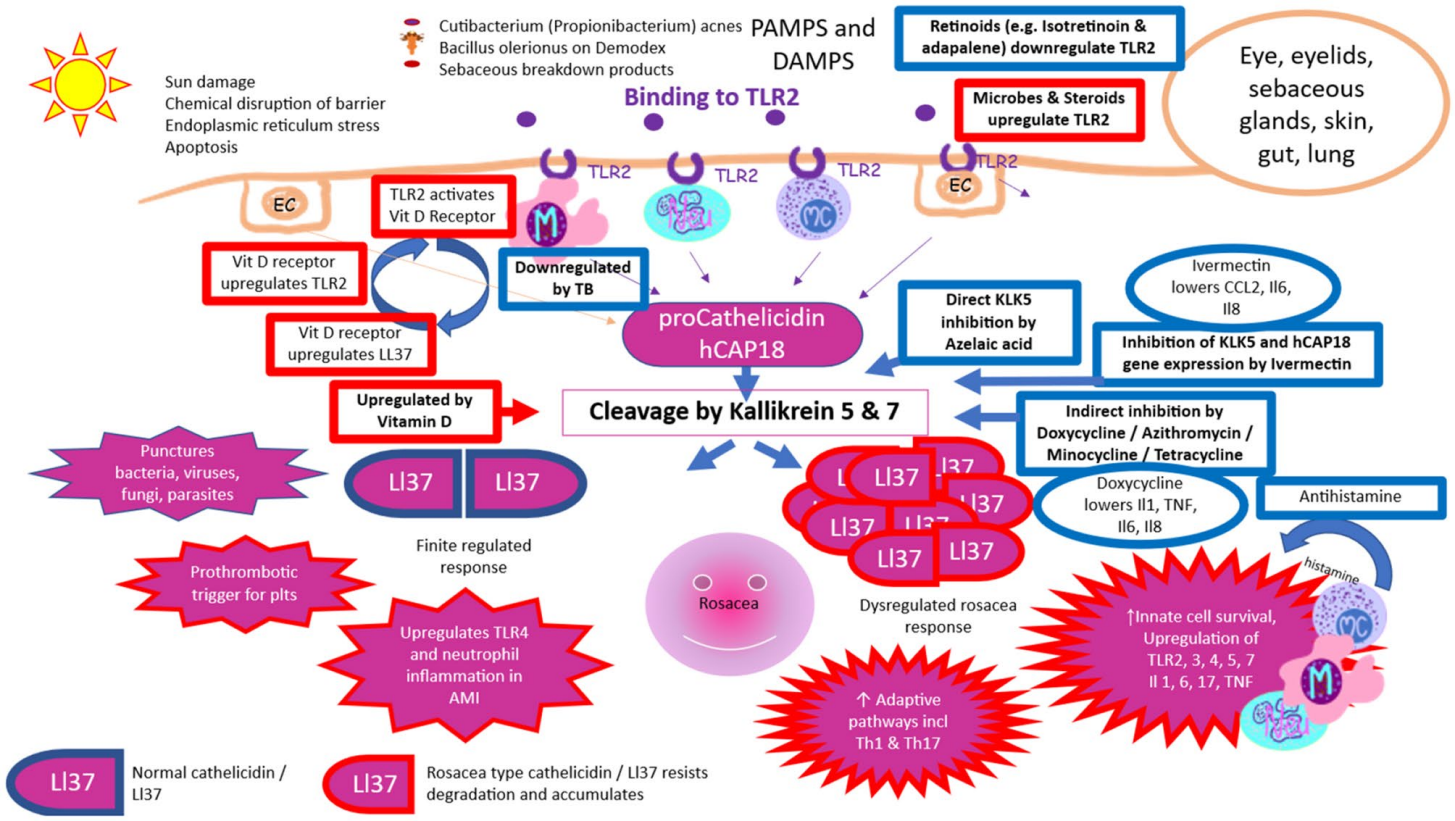
Innate immune components of the cathelicidin pathway showing dysregulation in the rosacea phenotype. Upregulation of the pathway is shown in red boxes and downregulation in blue boxes. hCAP18 (human cationic antimicrobial protein18 – cathelicidin precursor), KLK (kallikrein), M (monocyte-macrophage lineage cell), Neu (neutrophil), MC (mast cell), EC (epithelial or endothelial Cell), PAMP (pathogen associated molecular pattern), DAMP (damage associated molecular pattern), Il (Interleukin), TNF (Tumour Necrosis Factor), CCL2 (chemokine ligand 2), TLR (toll like receptor), AMI (acute myocardial infarction)

A 65-year-old obese diabetic woman with bilateral pseudophakia and glaucoma on travoprost, timolol and brinzolamide developed red eyes with bilateral mucopurulent conjunctivitis which did not respond to intensive topical ciprofloxacin and dexamethasone drops. By the time of referral she had developed bilateral beefy red injection with orbital ache waking her at night and SSS 8/25. Oral ibuprofen had been somewhat helpful in pain control. Lid margin telangiectasiae with periocular dermatitis and facial rosacea were noted and a diagnosis of rosacea scleritis was made. Travoprost was stopped and oral doxycycline 100 mg twice daily was started. Within 10 days both eyes were quiet and comfortable with SSS 0/25 and she had ceased non-steroidal anti-inflammatory treatment. The patient was happy that her skin was looking better than it had in years. After completing the remainder of 3 months on doxycycline 50 mg daily, her skin and eye inflammation remained in remission and she was able to re-commence her usual topical glaucoma medication including travoprost without significant side effects (Figs. 2 and 3).

### Case 4

A 69 year old woman presented on multiple occasions over several years to different members of an ophthalmology practice with conjunctivitis, marginal keratitis and episcleritis and was treated with topical chloramphenicol, topical steroid drops, and on occasion systemic non-steroidal anti-inflammatory treatment and standard advice about lid hygiene and lubricants. She tolerated the NSAIDs poorly because of dyspepsia. At her most recent presentation she had developed what was initially treated as episcleritis with oral celecoxib by her GP. She did not tolerate this and by the time she was referred for ophthalmic evaluation, her eye was beefy red and aching pain was waking her at night with SSS 8/25. She attributed the episode and others to the trigger factor of using mascara at the time of various special events. She was noted to have marked meibomian gland disease and her facial rosacea, previously concealed with make-up, was obvious. A diagnosis of rosacea scleritis was made. This settled to SSS 0/25 within 10 days on doxycycline 100 mg twice daily and she remained in remission after completing the remainder of 3 months on 50 mg doxycycline daily (Table 1).

**Table 1:** Clinical evolution and management prior to Doxycycline treatment

Patient	Ocular rosacea diagnosis	Systemic associations	Treatment helpful in reducing pain and inflammation but not in achieving remission	Ineffective treatment	Side effects of treatment
1	Sclero-uveitis	Type 2 diabetes Systemic vascular disease including CRVO	Topical antibiotics Topical steroids Systemic NSAID Systemic steroid (oral and pulsed IV)	Methotrexate Mycophenolate Cyclo-phosphamide	Glaucoma Cataract Renal cell carcinoma
2	Sclero-keratitis	Systemic vascular disease	Systemic NSAID	Topical antibiotics Topical steroids	
3	Scleritis	Type 2 diabetes Systemic vascular disease	Systemic NSAID	Topical antibiotics Topical steroids	
4	Episcleritis evolving to Scleritis		Systemic NSAID	Topical antibiotics Topical steroids	Dyspepsia

Abbreviations CRVO central retinal vein occlusion, NSAID Non steroidal anti-inflammatory drug

## Review and discussion

### Ocular rosacea pathogenesis and implications for ophthalmic management

A recent review of ocular rosacea for general ophthalmologists, succinctly summarises current understanding of cutaneous and milder ocular rosacea [9]. Shifting the perspective, this ophthalmic inflammation focused narrative review, discusses genetic and immunologic understanding. This has relevance, both to generalists and to subspecialists in ocular immunology, inflammation and infection who manage the more severe spectrum of disease including scleritis.

### Genetic and immunogenetic basis of ocular rosacea

A SNP (single nucleotide polymorphism), rs763035 has been identified in rosacea subjects in an immunologically relevant genetic area. It is intergenic and located upstream of the HLA-DRA gene and downstream of BTNL2 (butyrophilin-like 2, major histocompatibility complex class I associated). HLA-DRA (Human leukocyte antigen- DRA), a class II histocompatibility antigen, is found on antigen presenting cells and BTNL2 is a member of the immunoglobulin gene superfamily. The latter is associated with a number of human immunologically mediated diseases including inflammatory bowel disease and sarcoidosis. Variation in the HLA-DRA promoter region has been associated with multiple sclerosis. GWAS (Genome Wide Association Study) has shown association with HLA-DRB1*03:01, HLA-DQB1*02:01, and HLA-DQA1*05:01. These alleles have associations with type 1 diabetes, diabetic retinopathy, and coeliac disease. Together, these data strongly suggest a role for antigen presentation by HLA class II in the aetiology of rosacea [10]. Just as in keratoconjunctivitis sicca, elevated HLA-DR and ICAM-1 markers are found on the ocular surface of patients with ocular rosacea [11]. 

### Systemic associations

Meta-analysis has demonstrated a statistically significant association of rosacea with depression, hypertension, cardiovascular diseases, anxiety disorder, dyslipidemia, diabetes mellitus, migraine, rheumatoid arthritis, helicobacter pylori infection, ulcerative colitis, and dementia [12]. A case controlled study which assessed the severity of rosacea found moderate to severe rosacea to be significantly associated with hyperlipidemia, hypertension, metabolic diseases, cardiovascular diseases, and gastroesophageal reflux disease [13]. Blepharitis, arguably the commonest feature of ocular rosacea is an indicator of the onset of the metabolic syndrome [14]. Cathelicidin, in activating TLR4 signalling, has been shown to drive neutrophil related inflammation after acute myocardial infarction [15]. 

A key component of the rosacea pathway is LL-37 which is the only human cathelicidin. LL-37 is present at all human barrier surfaces and on white blood cells. It is part of the innate immune response to viruses [16] bacteria, and injury [17, 18], and has both vasoactive and pro-inflammatory activities [18]. It has been found to be elevated both in the serum and tissues of patients with cutaneous rosacea [19]. LL-37 is the product of cleavage of hCAP-18 (human cationic antimicrobial peptide-18), principally by kallikrein 5 and 7 (KLK5 and KLK7) [17]. In the non-rosacea phenotype, it is rapidly inactivated after performing its function. It has been shown that individuals with cutaneous rosacea express abnormally high levels of cathelicidin in their facial skin and that the persistent, proteolytically processed forms of cathelicidin peptides found in rosacea are different from those present in normal individuals [20]. Individual genetic and environmental factors determine downstream outcomes such as neovascularisation, neutrophil and mast cell activation or granuloma formation. Tear human neutrophil proteins 1–3 and conjunctival LL-37 gene expression rate have been found to be elevated in patients with oculocutaneous rosacea along with higher ocular surface disease index scores and lower tear break-up time [21]. Downstream enzymes such as Gelatinase B (MMP-9) and pro-inflammatory cytokines such as interleukin-1a, are elevated and may be involved in the pathogenesis of the irritation symptoms, recurrent epithelial erosions, vascularization, and epithelial basement membrane disorder that develops in the corneas of patients with this condition [22]. Even in the presence of elevated Il1 in ocular rosacea, TNF alpha has been found in one study to be normal on the ocular surface [23] although it has been found to be elevated in the skin during active facial rosacea [24]. 

A vitamin D response element has been found in the promotor of the cathelicidin gene [25]. Vitamin D and its analogues strongly induce cathelicidin / LL37 gene expression [25]. TLR (Toll like Receptor) activation of human macrophages up-regulates expression of the vitamin D receptor and the vitamin D-1-hydroxylase genes, leading to induction of LL37 and killing of intracellular Mycobacterium tuberculosis. It has been postulated that low vitamin D in dark skinned patients may increase susceptibility to diseases like tuberculosis [26] and possibly COVID-19 [27]. It has also been speculated that the high prevalence of rosacea in pale skinned people of Northern European extraction may relate to vitamin D induced upregulation of already supranormal LL37metabolites in the rosacea phenotype, resulting in ancestral resistance to tuberculosis and other diseases and conferring a survival advantage. This may account for the high prevalence of rosacea in these populations. In patients with rosacea, serum vitamin D can be lower than in normal controls, possibly because these patients avoid the sun [19]. Vitamin D levels in rosacea affected tissues, even where cathelicidin is elevated, may also be subnormal [19]. This suggests both vitamin D dependent and vitamin D independent pathways may be important. As yet no clinical study has been performed to determine whether vitamin D supplementation may trigger or exacerbate rosacea. Caution should be exercised in supplementing all patients with uveitis or inflammatory eye disease with vitamin D and clinicians should look out for signs of rosacea before considering this option.

The most studied and important pattern recognition receptor (PRR) in rosacea appears to be Toll Like Receptor 2 (TLR2) [24]. TLR2 is elevated in ocular rosacea and results in increased expression of kallikrein 5 [24]. Microbial stimulation of kallikrein 5 using Cutibacterium (Propionibacterium) acnes is dependent on this TLR2 pathway [24]. Glucocorticosteroids also increase TLR2 expression in the presence of P Acnes [28, 29] or in the presence of inflammatory conditions where TNF is elevated [29]. It is possible that this mechanism is particularly important in rosacea scleritis as patients with scleritis are frequently started on steroid treatment by general ophthalmologists who may not connect the rosacea features with the scleritis. It is postulated that elevated TLR2 secondary to steroid treatment may be potentiating the rosacea cascade and worsening the scleritis. This interesting mechanism by which steroid treatment potentiates TLR2 related activity may also be relevant to other antigen related diseases such as Sarcoidosis [30–32] and Giant Cell Arteritis [33] which typically require very slow steroid taper once control is achieved.

### Local and systemic treatment of ocular rosacea including scleritis

Current guidelines for the management of ocular rosacea such as the 2022 S2k recommendations are based on expert opinion and apply to milder lid and ocular surface disease as well as to prevention of disease flares. Strategies with moderate to low levels of evidence include lid hygiene to reduce microbial triggers, lipid-based lubricants to prevent desiccation driving inflammation and anti-inflammatory treatment including topical cyclosporine or topical ivermectin as steroid sparing agents. The role of intense pulsed light and meibomian gland heat, probing and compression is an emerging area of interest. Systemic treatment with doxycycline or azithromycin / other macrolides are options for more severe disease and mention is made of systemic metronidazole and ivermectin, noting no good evidence supporting their use [34]. Within this S2k set of guidelines is a clear recommendation that glucocorticosteroids are not indicated for rosacea and that they may exacerbate it [34]. 

For severe disease including scleritis the use of doxycycline is supported by clinical evidence and increasingly through understanding of its immunological mechanisms. Doxycycline indirectly inhibits KLK5, a serine protease subject to regulation by matrix metalloproteinases (MMPs), resulting in reduced conversion of procathelicidin (hCAP18) to cathelicidin (Ll-37) [35]. This appears to be via non-specific MMP inhibition [36]. MMP-9 and TIMP-1 (tissue inhibitor of matrix metalloproteinase) are produced by the human corneal epithelium and are present in tear fluid. MMP-3 alone is sufficient to activate MMP-9 on the ocular surface. Doxycycline does not directly inhibit this activation by MMP-3, but it decreases MMP-9 activity in cell culture [37]. MMP-8 (collagenase-2) concentration and degree of activation in tear fluid are increased in ocular rosacea, probably reflecting increased inflammatory activity. Doxycycline 100 mg daily for 4 weeks followed by 50 mg daily for 4 weeks effectively reduces these pathologically excessive levels and activation of MMP-8, and relieves patients’ subjective symptoms [38]. By downregulating degradation resistant rosacea type cathelicidin, doxycycline lowers inflammatory drivers [22, 23, 39, 40] such as Il1, TNF, Il6, Il8, Il17 and histamine. Adaptive pathways related to Th1 and Th17 cells as well as activated innate cells such as neutrophils, monocyte-macrophage cells and mast cells are no longer subject to rosacea related upregulation. In this way doxycycline is targeting many of the same pathways as systemic steroids and other csDMARDS and bDMARDs (conventional synthetic and biological disease modifying antirheumatic drugs). Its particular advantage is that it does not upregulate TLR2 in the presence of microbes / TNF in the same way that steroid treatment does. This is particularly important in the setting of rosacea scleritis where lid margin factors such as commensal and non-commensal microbes bind TLR2, acting as a trigger and perpetuator of inflammation. Conventional treatment of undifferentiated scleritis might include systemic steroids which, while exerting a broad- spectrum anti-inflammatory effect, in the setting of rosacea, simultaneously have the undesirable side effect of upregulating the entire downstream inflammatory pathway mediated by TLR2, potentiated by the nature of the microbially populated lid margin.

Numerous topical and systemic treatments form part of established and experimental treatment of rosacea [34, 41]. To date, evidence to support preferential use of doxycycline, minocycline, azithromycin or other similar agent in ocular disease is limited by lack of prospective, controlled comparative evidence as well by incomplete understanding of effective dosing regimens. Doxycycline may be preferable because of its lower risk of slate grey pigmentation and raised intracranial pressure than minocycline, lower risk of cardiac side effects than minocycline or azithromycin and lower risk of microbial resistance than full dose azithromycin. In addition, controlled release, low dose (sub-antimicrobial) doxycycline 40 mg is available in some countries including the USA, further reducing the risk of antimicrobial resistance. Photosensitisation and gastrointestinal side effects may limit its use in some people and patients need to be reminded that its absorption may be reduced by simultaneous consumption of dairy products (although this is less of an issue than with tetracycline).

For patients unable to tolerate doxycycline and related medications, it is unclear what alternatives would be best in the setting of episcleritis / scleritis. Non- steroidal anti-inflammatory drugs in this small series did appear to ameliorate pain to some extent but did not result in remission of scleritis. Topical and systemic retinoids such as isotretinoin and adapalene downregulate TLR 2 [42]. Systemic retinoids could logically be used to prevent rosacea pathways being potentiated by steroid treatment. Ivermectin used topically for rosacea may both eliminate demodex and directly downregulate KLK-5 and Cathelicidin gene expression [43]. Systemic treatment with ivermectin has had very limited use in the management of facial rosacea or rosacea and its side effect profile is a limiting factor. Like retinoids, it could potentially be worthy of consideration in severe disease where conventional treatment was not tolerated.

### Application of current understanding to this patient series

Diseases known to have systemic association with rosacea were seen in our patients. These included diabetes, metabolic syndrome and vascular occlusion.

All patients had received, topical, oral or intravenous steroid treatment or non-steroidal anti-inflammatory treatment prior to therapy being modified in tertiary ocular inflammation clinics because of suboptimal efficacy of these treatments. One patient experienced steroid induced glaucoma and worsening of diabetic control on steroid therapy as well as renal cell carcinoma following cyclophosphamide treatment. Steroid treatment may have worsened rosacea related inflammation in the patients with the most severe inflammation by upregulating TLR2 in the presence of microbial triggers [28, 29]. Patients in whom systemic steroid treatment was avoided experienced rapid and uneventful remission. Standard initial management of most types of severe ocular inflammation includes steroid treatment and these cases illustrate that careful exclusion of rosacea may be advisable before starting systemic steroid treatment. Future studies will need to evaluate the positive or negative role of topical steroids in this type of inflammation. Timing may be key, with priority given to inhibition of TLR2-Ll37 pathways prior to use of steroid treatment.

While topical antimicrobial therapy may have a role to play in minimising microbial upregulation of TLR2, it was ineffective in the initial management of all of our patients. Commencement of doxycycline was followed by rapid disease control in all cases. The likely principal mechanism of action was through its non-specific effect on matrix metalloproteinases blocking kallikrein-5 and reducing conversion of procathelicidin (hCAP-18) to cathelicidin (Ll17). One patient required long-term treatment in order to maintain remission. No patient experienced intolerable side-effects and no other similar drugs were substituted. To date there is no clarity on optimal initial dosing. Logically it would be reasonable to deduce that given upregulation of TLR2 in these cases, most of which have been treated with steroids prior to the rosacea being recognised, a high level of MMP blocking is needed initially. This would justify an initial dose of at least 100 mg daily or possibly double this dose in severe cases.

Vitamin D was not evaluated in this series but the role of vitamin D analogues and receptors in potentiating rosacea pathways is an important factor to consider in future studies. Given that early evidence supports the role of vitamin D in controlling non-infective uveitis [44], future studies need to pre-emptively identify patients with rosacea who may confound the results of prospective investigations.

## Conclusion / Important messages

- It is vital to recognise the features of ocular rosacea in the setting of scleritis or episcleritis as up to 15% of patients with rosacea have only ocular manifestations without facial features.
- Specific treatment for rosacea in the form of doxycycline or related drugs may be safer, work very quickly, have fewer side effects and be less expensive than agents used for non-rosacea scleritis.
- Steroid and vitamin D treatment have the potential to adversely affect the management of rosacea scleritis and episcleritis through potentiation of molecular pathways upregulating cathelicidin.

## Data Availability

No datasets were generated or analysed during the current study.
